# Potential cellular endocrinology mechanisms underlying the effects of Chinese herbal medicine therapy on asthma

**DOI:** 10.3389/fendo.2022.916328

**Published:** 2022-08-16

**Authors:** Zeyu Meng, Huize Chen, Chujun Deng, Shengxi Meng

**Affiliations:** ^1^ The Second Clinical Medical College, Heilongjiang University of Chinese Medicine, Harbin, China; ^2^ Department of Traditional Chinese Medicine, Shanghai Jiao Tong University Affiliated Sixth People’s Hospital, Shanghai, China

**Keywords:** cellular endocrinology, neuroendocrine, pulmonary neuroendocrine cell, cellular pathways, hormonal response, asthma, traditional Chinese medicine, mechanisms

## Abstract

Asthma is a complex syndrome with polygenetic tendency and multiple phenotypes, which has variable expiratory airflow limitation and respiratory symptoms that vary over time and in intensity. In recent years, continuous industrial development has seriously impacted the climate and air quality at a global scale. It has been verified that climate change can induce asthma in predisposed individuals and that atmospheric pollution can exacerbate asthma severity. At present, a subset of patients is resistant to the drug therapy for asthma. Hence, it is urgent to find new ideas for asthma prevention and treatment. In this review, we discuss the prescription, composition, formulation, and mechanism of traditional Chinese medicine monomer, traditional Chinese medicine monomer complex, single herbs, and traditional Chinese patent medicine in the treatment of asthma. We also discuss the effects of Chinese herbal medicine on asthma from the perspective of cellular endocrinology in the past decade, emphasizing on the roles as intracellular and extracellular messengers of three substances—hormones, substances secreted by pulmonary neuroendocrine cells, and neuroendocrine-related signaling protein—which provide the theoretical basis for clinical application and new drug development.

## Introduction

Asthma is a pulmonary disease with limited trachea characterized by reversible airflow obstruction and chronic complex inflammation, which easily worsens and more often affects female than male patients (1, 2). In addition to airway inflammation, airway remodeling and hyperreactivity also contribute to the pathophysiology of asthma (3, 4). The underlying mechanisms of asthma pathogenesis have not been fully elucidated. It is currently accepted that the mechanisms of airway immunity–inflammation and neuroregulation are the important pathogenesis of asthma (5). It is estimated that 300 million individuals worldwide suffer from asthma, with a projected increase of an additional 100 million individuals by 2025 (6, 7). Substantial morbidity and annual healthcare expenditure place an immense burden on individuals and society (8).

Currently, the use of inhaled glucocorticoids (GCs) is one of the most effective anti-inflammatory therapies used for the treatment of asthma (9). However, up to 30–50% of asthmatic patients are hyporesponsive to corticosteroid treatment (10). The subtypes of severe asthma represented by steroid-resistant asthma, steroid-dependent asthma, account for 63% of the annual total medical costs for treating asthma (11). Short-acting beta-2 agonists (SABA) represented by salbutamol and terbutaline, long-acting beta-2 agonists (LABA) represented by salmeterol, and formoterol are also commonly used in the treatment of asthma. In German (12), Swedish (13), and Chinese (14) population-based studies, the overuse of SABA is associated with an increased risk of asthma exacerbation and mortality. In another study, the overuse of SABA may cause adverse reactions such as hypokalemia, tachycardia, transient hypoxemia, and hyperglycemia (15). LABA/long-acting muscarinic antagonist (LAMA) combination agonist is widely used in the treatment of asthma (16). However, this combination has some potential pharmacological risks like cardiac arrhythmia (17). Currently, traditional Chinese medicine (TCM), owing to their unique curative effects without the development of significant toxic side effects, has been widely applied in the treatment of asthma in China (18). Thus, under the guidance of cellular endocrinology, it has gradually attracted the attention of researchers for known effective TCM extracts, single herbs, traditional Chinese patent medicine, and compound prescriptions for the treatment of asthma to be selected and for the underlying molecular mechanisms to be revealed.

The underlying cellular and molecular mechanisms for the effects of TCM in treating asthma have been extensively studied in recent years—for example, curcumin can inhibit the proliferation and differentiation of tracheal epithelial cells through the NF-κB/iNOS/COX-2 signal pathway, inhibit smooth muscle cell proliferation through the Wnt/β catenin signal pathway, exert an antioxidant effect through Nrf2/HO-1, and affect cell cycle and cytokine signal transduction through the Erk-p38-JNK pathway. Nevertheless, there is still a lack of in-depth, systematic, and inductive research on TCM from the perspective of cellular endocrinology (19).

In this review, three aspects will be classified in terms of the mechanisms of TCM in the treatment of asthma from the perspective of cellular endocrinology in order to provide guidance for asthma treatments and insights into related inflammatory immune mechanisms.

## Substances secreted by pulmonary neuroendocrine cells act as messengers in the treatment of asthma with TCM

**Figure 1:**
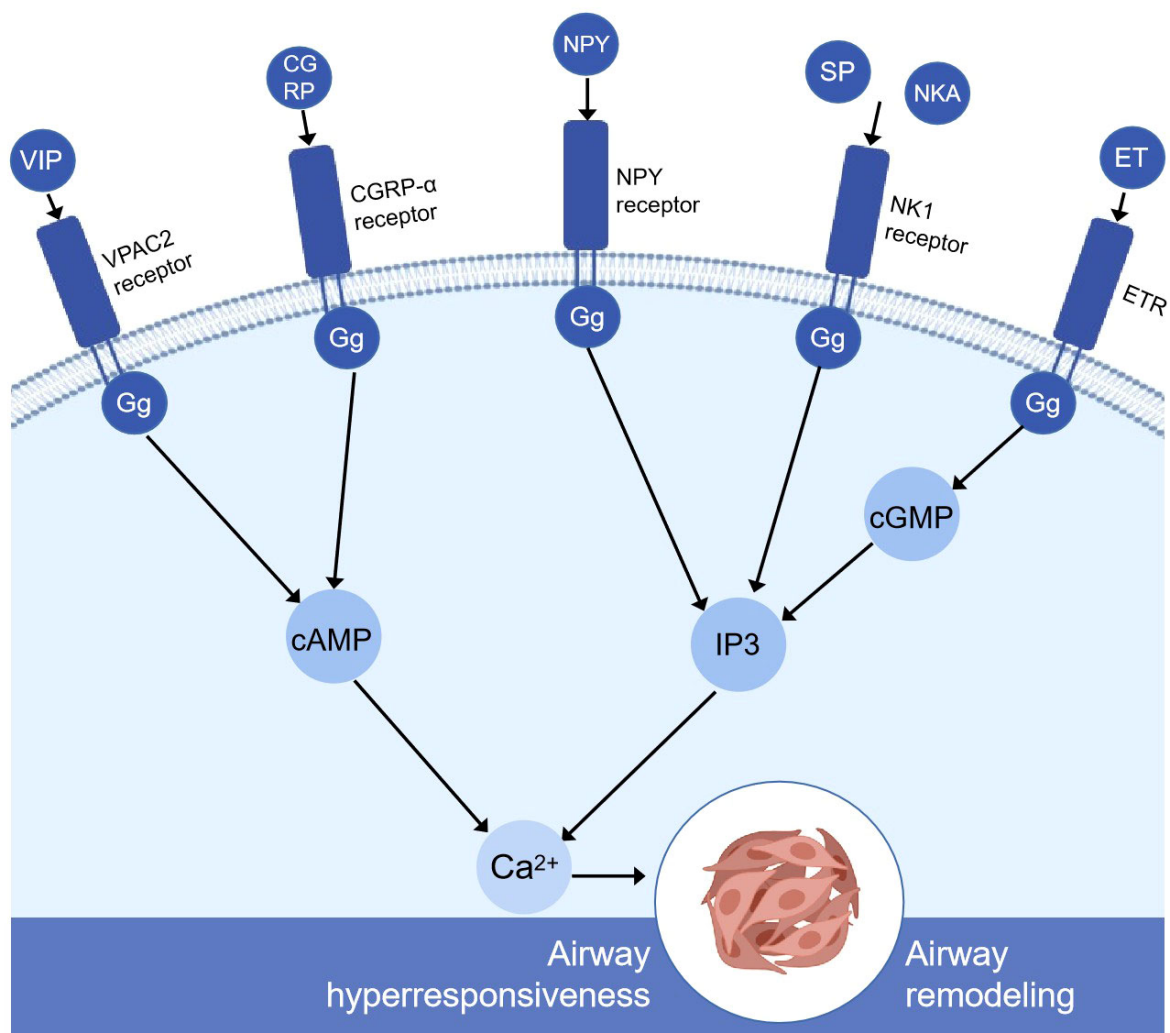
Simplified schematic diagram of the molecular mechanism of pulmonary neuroendocrine cell secretion-mediating airway remodeling and airway hyperresponsiveness. VIP, CGRP, and G protein-coupled receptors increase cAMP through adenylyl cyclase (not shown). Then, cAMP increased intracellular calcium through protein kinase A (not shown). NPY, SP, NKA, and G protein-coupled receptors altogether cause the breakdown of inositol 1,4,5-phosphate (IP3) and the subsequent release of calcium ions from intracellular storage. ET is produced by the stimulation of protein kinase C (not shown), binds to other receptors in lung tissue, such as the endothelin receptor, activates cyclic GMP, secondary to increased IP3 levels, and releases the calcium ions stored in the cells. High levels of calcium can severely affect airway smooth muscle cells, airway epithelial cells, and goblet cells and ultimately lead to airway remodeling and airway hyperresponsiveness. VIP, vasoactive intestinal peptide; CGRP, calcitonin gene-related peptide; NPY, neuropeptide Y; SP, substance P; NkA, neurokinin A; ET, endothelin; ETR, endothelin receptor; VPAC2, vasoactive intestinal peptide receptor 2; IP3, inositol 1,4,5-triphosphate; cAMP, cyclic adenosine monophosphate; cGMP, cyclic guanosine monophosphate.

Pulmonary neuroendocrine cells (PNEC) are a kind of neurosensory cells sparsely distributed in the nasal respiratory tract epithelium, pharyngeal mucosa, and entire respiratory tract from the trachea to the terminal bronchioles. They are isolated cells or aggregates in the airway epithelium. The aggregated PNEC is called neuroepithelial body (NEB) (20) and is located in the innervation cluster at 20–30 cells (21). PNEC accounts for about 1% of the total number of airway cells (22). Although it has a small number in lung tissue, it can be used as a messenger and a chemical sensor. There are studies showing that PNEC can convert exogenous airway signals such as pollutants, bacteria, virus, and allergens into downstream cascade reactions *in vivo*, releasing dense vesicles rich in vasoactive peptides, substance P, calcitonin gene-related peptide, neurokinin A, neurokinin B, neuropeptide Y, endothelin, and some neurotransmitters (23). These bioactive molecules secreted by PNEC are closely related to a variety of lung diseases such as asthma (24, 25). They can regulate the response of adjacent airway smooth muscles and then regulate the airway immune response (26, 27) (**Figure 1**).

### Vasoactive peptide

Vasoactive intestinal peptide (VIP), which has powerful anti-inflammatory effects, is a neuroendocrine and immunopeptide produced by activated T cells and synaptic nerves, such as cholinergic and sensory nerves (28). It is one of the most abundant bioactive peptides involved in the control of both inducing and promoting type 2 immune responses in human lungs (29). Moreover, it can both dilate blood vessels to improve cardiopulmonary blood circulation and dilate bronchi to regulate airway secretion (30).

**Figure 2:**
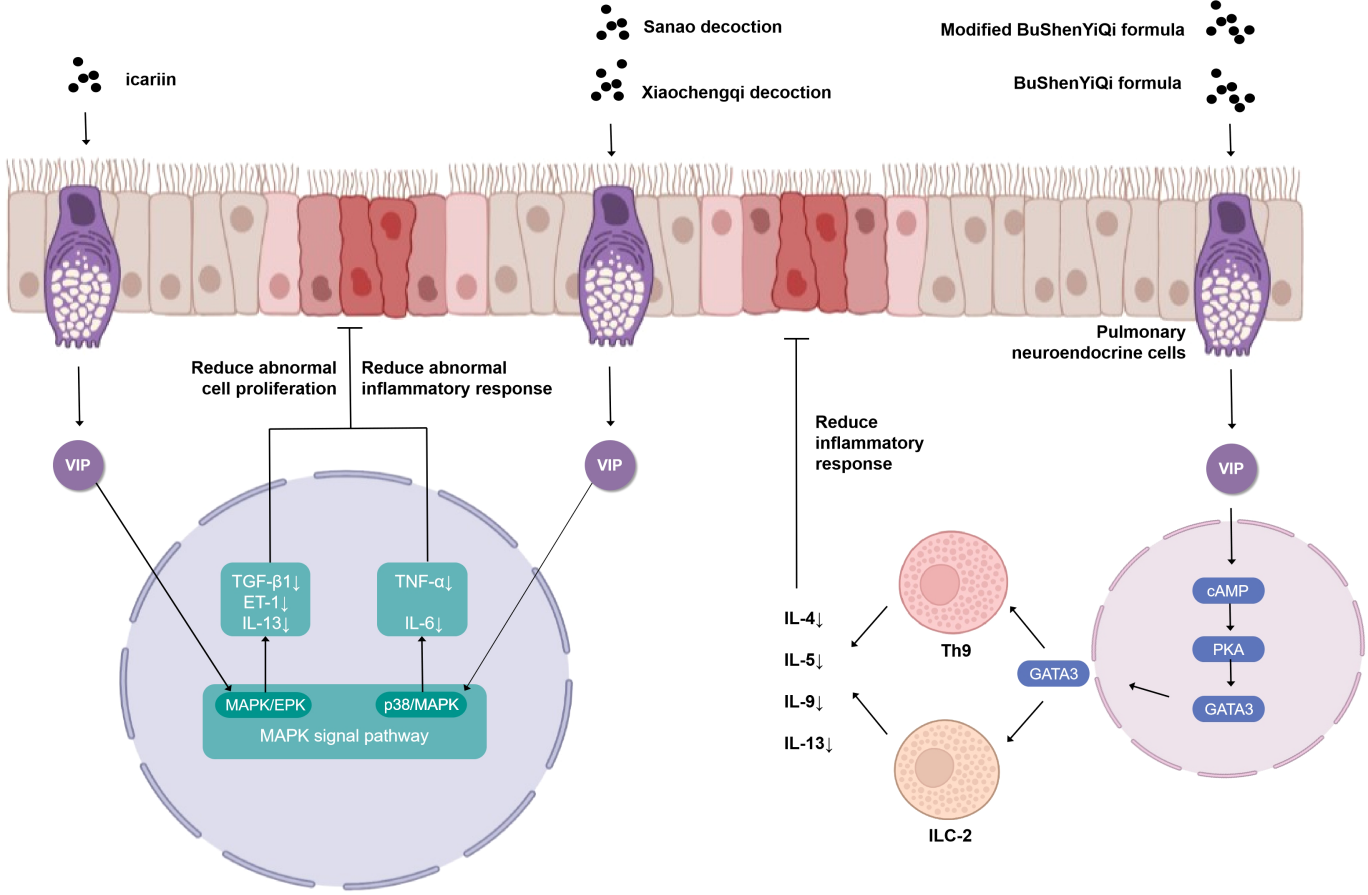
Simplified schematic diagram of cellular endocrine mechanism of vasoactivepeptide in the treatment of asthma with traditional Chinese medicine.

The VIP plays a prominent role in the study of the cellular endocrine mechanism of TCM in the treatment of asthma (**Figure 2**). Upon antigen stimulation, macrophage cells, T cells, lung type 2 innate lymphoid cells (ILC2s), and other immune cells promote the production of VIP and autocrine or paracrine by other cells in the lung (31). Then, the VIP binds to G protein-coupled receptor VPAC2 receptor to increase intracellular cyclic adenosine monophosphate (cAMP) by stimulating adenylate cyclase activated by protein kinase A (PKA) (32, 33). IL9 and GATA binding protein 3 (GATA3) are produced through this cAMP-dependent pathway, and GATA3 can further induce type 2 cytokines (IL-13, IL-9, and IL-5) that activate ILC2, Th9 cells, and Th2. The BuShenYiQi formula reduced the content of ILC2 and Th9 and the type 2 cytokine (IL-13, IL-9, IL-5) of Th2 by inhibiting the VIP-cAMP-PKA-GATA3 signal pathway. It means that the BuShenYiQi formula alleviates airway inflammation and mucus oversecretion by blocking the expansion and differentiation of ILC2 and Th9 cells, thus alleviating the progression of asthma (33). There are abundant glycosides and flavonoids and some iridoid glycosides in the BuShenYiQi formula, such as catalpo, icariin, lbaicalin, albiflorin, and paeoniflorin. These monomers of TCM may have cumulative or synergistic effects on asthma, which provides a direction for TCM to treat asthma through VIP in the neuroendocrine pathway.

Similar to the BuShenYiQi formula, the modified BuShenYiQi formula reduces the expression of VIP and the percentage of ILC2 and Th9 cells (31) through the VPAC2-cAMP-PKA-GATA3 signaling pathway and reduces the content of Th2 inflammatory cytokines (IL-4, IL-5, and IL-13) (34) through another pathway, which jointly alleviates mucus oversecretion and airway inflammation.

The combination of Sanao decoction and Xiaochengqi decoction not only promoted the release of VIP from lung tissue but also promoted the release of VIP from the intestine, which entered the lung tissue through the blood circulation, thus increasing endogenous VIP and thereby inhibiting the over-activation of the p38MAPK signal pathway, inhibiting the activation of immune cells, especially alveolar macrophages, reducing the synthesis and release of inflammatory factors TNF- α and IL-6, and thus significantly reducing the increase of airway epithelial goblet cell metaplasia and mucus hypersecretion in the lumen in order to treat asthma (35). Modified Xiaofeng San can increase the content of VIP in plasma and improve the related indexes of lung function obviously (36).

### Substance P

Substance P (SP) is an undeceptide secreted mainly by neurons and is a member of the family of tachykinins (37, 38). The importance of SP in information transfer between cells through paracrine or endocrine signaling is well established (39). SP has shown potent contraction of airway smooth muscle and promotion of plasma leakage properties owing to its biological and functional properties (40). It has been demonstrated that some immune cells have also been found to secrete SP, suggesting that it plays an indispensable role in immune response, such as chemotaxis of monocytes and eosinophils, degranulation of mast cells and eosinophils, enhancement of leukotrienes, and so on (41). Moreover, SP can also bind to its selective receptor neurokinin-1 receptor (NK-1R) *via* G-protein-coupled receptor pathway and exert a variety of biological effects (42). Airway epithelial injury in asthmatic patients exposes the endings of pulmonary nerve fibers (43), and the stimulation of inflammatory mediators (44) leads to the secretion of SP in the airway *via* the mechanism of axonal reflex (45). SP can specifically induce human bronchial epithelial cells to synthesize chemokines (CCL4, CCL5, IL-6, IL-8, TNF-α, IL-31, IL-33, and vascular endothelial-derived growth factor) (46–48), which further cooperates with the progression of asthma inflammatory response.

LgE can activate mast cells, eosinophils, and cells involved in antigen presentation in the body, causing mast cells and eosinophils to degranulate and release inflammatory mediators such as leukotrienes, thus triggering an inflammatory response to asthma (49). When the SP and calcitonin gene-related peptide (CGRP) content of airway epithelial cells increased, airway smooth muscle contraction, glandular secretion, and also stimulated lgE secretion increased. VIP has an antagonistic relationship with SP and CGRP, which can not only relax the smooth muscle but also reduce the level of lgE. Sinapis alba San can effectively improve allergic asthma by increasing the VIP in serum, weakening the expression of SP and CGRP in lung tissue, and significantly reducing the level of lgE (50).

**Figure 3:**
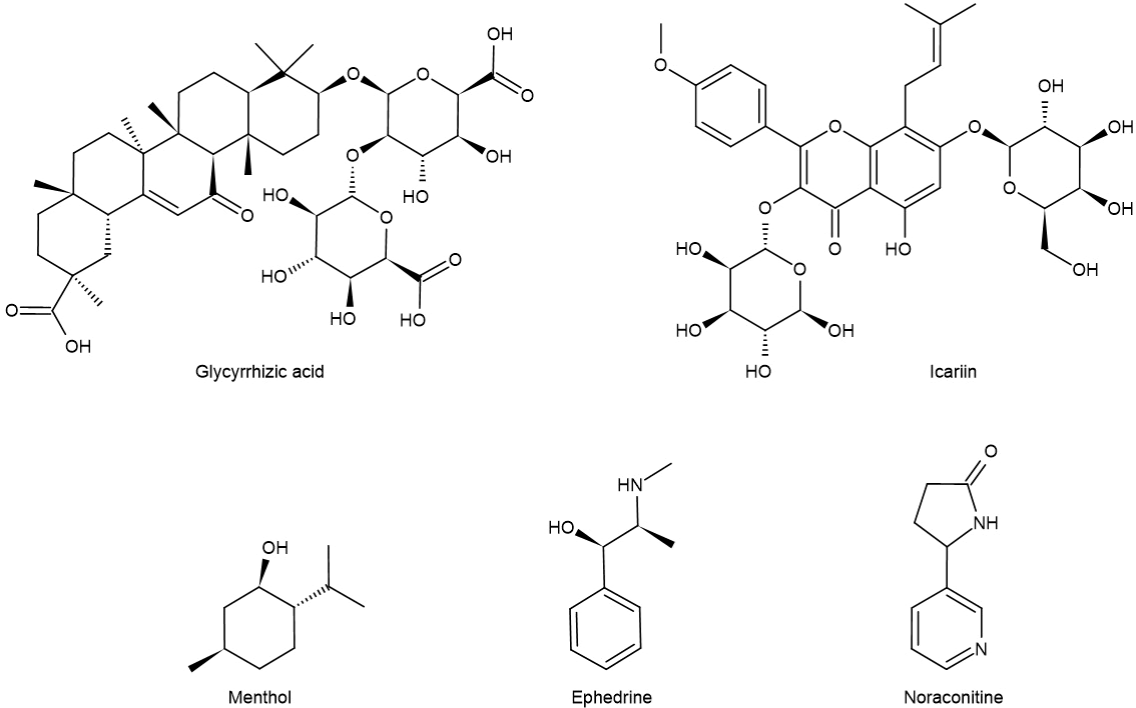
Chemical formula of some monomers of traditional Chinese medicine for the treatment of asthma.

Maxing Shigan decoction can significantly reduce the levels of IL-4, IL-13, PGE2, and SP in bronchoalveolar lavage fluid (BALF) by downregulating TRPV1 protein, decreasing the expression of epidermal growth factor receptor in the trachea, and decreasing the expression of IL-2 and TNF-α (51) to repair the pathological changes of tracheal tissue in different degrees. Yupingfeng prescription can treat cough variant asthma in children by reducing the levels of IL-5 and SP in serum (52). In addition, menthol (formula: C_10_H_20_O) (**Figure 3**) attenuates airway inflammation and airway hyperresponsiveness in asthmatic mice, which may be related to the decrease of SP and NK-1R expression in bronchial epithelial cells (53, 54). Modified Xiaofeng San (36) can reduce the content of SP in plasma and significantly improve the related indexes of pulmonary function such as FEV1, FEV1/FVC, and FEV1%.

### Calcitonin gene-related peptide

Calcitonin gene related peptide is a 37-amino-acid protein of the calcitonin peptide family, which is secreted by central and peripheral neurons and acts on inflammatory cells to induce the release of inflammatory mediators (55). The distribution location of CGRP is different; it causes neurogenic inflammation, and its specific role in the occurrence and pathogenesis of asthma is also different. When distributed in the airway epithelium, it can induce epithelial differentiation; when distributed in airway vessels, it can dilate blood vessels; when distributed in airway smooth muscles, it can contract airway smooth muscles (56). The high expression of CGRP in patients will cause inflammation and airway hyperreaction, which is not conducive to the recovery of asthma.

Similar to the molecular mechanism of VIP production and interaction, after nerve fibers extend into the airway, pulmonary neuroendocrine cells activated by Ca^2+^ flow release CGRP to lung tissue. GPCR binds to G protein, increases intracellular cAMP level, and activates the cAMP signal pathway, activating PKA. PKA further regulates the phosphorylation level of many transcription factors (57). After the nerve fibers extend into the airway, the pulmonary neuroendocrine cells release CGRP into the lung tissue. CGRP can stimulate the degranulation of mast cells such as ILC2 as IL-33 is directly stimulated by allergens and induces a downstream immune response to produce IL-5 and IL-13. To some extent, CGRP can amplify the effect of allergic asthma (58). While the vagus nerve innervating the airway releases CGRP through pulmonary C fibers, it also releases acetylcholine, which can regulate the activity of ILC2 after binding to α 7 nicotinic acetylcholine receptor and neuropeptide receptor, respectively (59). Maxing Shigan decoction may inhibit the release of CGRP, reduce airway sensitivity, and improve airway inflammation by inhibiting the expression of TRPV1 (60). It is well established that Huanglong cough oral liquid can reduce the levels of airway neurogenic inflammatory mediators CGRP, leukotriene E4, and nerve growth factor (NGF) and achieve the effect of prevention and treatment of asthma (61).

### Neurokinin A and neurokinin B

Just like SP, neurokinin A (NKA) and neurokinin B (NKB) belong to the tachykinin family. NKB is a polypeptide encoded by Tac2 encoded by Tac1 (62). NKA and NKB are also endogenous ligands for NK2 receptors which are distributed in the pharynx, larynx, trachea, bronchi, and lung organs (63). NkA is responsible for transmembrane electrochemical gradients, determining intracellular ion homeostasis, metabolite transport, and regulation of intercellular and intracellular signals; so, its downregulation is related to the formation of many diseases, including asthma and allergic diseases (64). Most of the studies have shown that the increase of respiratory SP and NKA levels is closely related to chronic obstructive pulmonary disease (COPD) and asthma (65, 66). The researchers found that interferon-γ did not cause neutropenia; however, it could replace Th1 cells to cause an increase in AHR and significantly induce the production of NKA and the expression of neurokinin-2 receptor (NK2R) in the lung. NK2R antagonist can significantly inhibit the increase of interferon-γ-dependent AHR in OVA-induced asthmatic mice, and there is no significant change in the expression of NK2R mRNA. In addition, it can also reduce the influx of Ca^2+^. Therefore, these results reveal an interesting molecular mechanism of neuroendocrine immunology associated with asthma: interferon-γ directly acts on airway smooth muscle cells (ASMC), acts on ASMC through the NKA/NK2R signal cascade, and increases AHR (64).

Researchers undertook a study about mirabilite. Following modeling, asthmatic mice were administered by gastric gavage with mirabilite to stimulate the large intestine. The results showed that VIP was highly expressed in both lung and intestinal tissues, while the expression level of SP in lung tissue and intestinal tissue was the opposite, and the content of SP in lung tissue decreased. Similarly, the expression level of NK-1R in lung tissue and intestinal tissue was the opposite, and the expression of NK-1R in lung tissue tended to be low. The expression levels of NKA and NKB in lung tissue and intestinal tissue were similar, and the expression degree was decreased. It can be seen that mirabilite stimulation of the intestinal tract can transmit the stimulation to the lung by way of the neuroendocrine and then regulate the secretion of VIP, SP, and receptors in the lung tissue (67). This explains the TCM theory of the “lung and large intestine stand in interior–exterior relationship” from the perspective of the neuroendocrine, which was first recorded in The Yellow Emperor’s Inner Canon) and is a classical basic theory of traditional Chinese medicine (68–70). At present, the research on the relationship between lung and intestinal axis (71–73) and the relationship between gut and respiratory tract (74, 75) also confirm this theory to some extent. While modified Bainiu Xuanfei decoction can reduce NKA, SP, TNF-a, IL-8, IL-4, IL-5, and CGRP in lung tissue (76), Minke Jian can reduce SP and NKA in the supernatant of induced sputum and related cytokines such as IFN-γ, TNF-α, IL-4, and IL-5 in plasma (77). The Huatan Huoxue formula is a combination of Sanzi Yangqin decoction and Taohong Siwu decoction. It can reduce the total IgE in serum, the content of inflammatory factors IL-4, IL-9, and IL-13 in BALF, the content of IL-25, IL-33, TSLP, IL-6, and AREG related to ILC2s, and ILC2s at the same time. In addition, it can reduce the content of iILC2s and nILC2s in the small intestine and the level of iILC2s in lung tissue but has no significant effect on the production of nILC2s in lung tissue. The mechanism of action is closely related to the lung–intestinal axis, and it is also related to blocking the migration of iILC2s from the small intestine to the lung and reducing the content of ILC2s in the lung tissue, thus reducing the type 2 immune response (78).

### Neuropeptide Y

Neuropeptide Y (NPY) is a 36-amino-acid peptide, which is the most abundant neuropeptide in the brain. It is mainly distributed in the central nervous system, such as the cerebral cortex, hippocampus, thalamus, hypothalamus, and brainstem (79). Its c-terminal sequence ends with tyrosine (Y), so it is named neuropeptide Y (80). NPY can couple with G protein and activate Y receptor (Y1–Y6) (81). The co-release of NPY and norepinephrine can play a role similar to VasculartoneNO and participate in the regulation of vascular tension (82). Research has indicated that the loss of NPY and NPY-Y1 receptor signals can protect the mice from airway inflammation and hypersensitivity. It has been proved that NPY aggravates the progression of allergic asthma mainly through NPY-Y1 receptors expressed by T cells, eosinophils, and other immune cells (83). In addition, during the acute attack of asthma, the level of NPY was positively correlated with airway hyperresponsiveness (84). Other studies suggest that NPY may be treated by coursing the liver and resolving depression through the brain–gut axis and emotional diseases such as psychological stress asthma (85–87).

### Endothelin

Endothelin (ET) is a kind of peptide with 21 amino acids, which is mainly secreted by airway epithelial cells and pulmonary neuroendocrine cells (88). ET-1, ET-2, and ET-3 are all members of the ET family. ET-degrading enzymes are mainly located in the lungs and kidneys, so the lung tissue is also an important site for clearing and decomposing ET (89). Studies have confirmed that ET-1 is closely related to airway remodeling in bronchial asthma. ET-1 and their precursors exist in airway epithelial cells and submucosal glands, and the process of stimulating ET-1 synthesis requires the participation of Ca^2+^ and dependent protein kinase C. ET-1 binds to other corresponding receptors such as endothelin receptor in lung tissue and activates the second messenger cyclic guanosine monophosphate. The secondary increase of inositol triphosphate level induces the increase of intracellular Ca^2+^ and exerts its biological effect (88).

IL-13 is a Th2-type cytokine produced by activated mast cells, T cells, and basophils. Allergens can regulate ASMC through IL-13 to lead to airway hyperresponsiveness (90). In addition, IL-13 has been shown to cause bronchial smooth muscle hyperplasia and increased mucus secretion through the c-Jun kinase/STAT pathway. IL-13 can also inhibit the secretion of pro-inflammatory mediators from monocytes and macrophages, including PGs, intermediates of reactive oxygen species, and nitrogen, IL-1, IL-6, IL-8, TNF-α, and IL-12 by inhibiting the mechanism of NF-κB (91, 92). Like IL-13, ET-1 has the property of promoting cell proliferation. ET-1 can inhibit the apoptosis of ASMC, promote the division of ASMC, and induce the proliferation of ASMC. Icariin (formula: C_32_H_38_O_16_) (**Figure 3**) reduces TGF-β1 and VEGF by inhibiting the release of ET-1 and IL-13 and inhibits the proliferation of ASMC by inhibiting the MAPK/Erk signal pathway, thus alleviating the degree of airway remodeling in asthma (93).

## Hormones act as messengers in the treatment of asthma with TCM

### Dopamine

Dopamine (DA), as a predominant catecholamine neurotransmitter, was widely distributed in the central nervous system (94, 95). It is a key neurotransmitter in the hypothalamus and pituitary and secreted by specific neurons in a certain part of the brain (96). The DA receptor is a member of the G protein-coupled receptor family (97, 98), which is divided into two families: D1-like receptors (D1 and D5) and D2-like receptors (D2, D3, and D4) (99, 100). L-741626, a dopamine D2-like receptor antagonist, is expressed in airway smooth muscle and can relax ASM, which further indicates that dopamine D2-like receptor induces bronchiectasis by activating the intracellular cAMP signal pathway (101), while some researchers noticed that SCH23390, a dopamine D1-like receptor antagonist, had a contractile effect on the trachea and then found that there were DRD1 receptors in rat airway smooth muscle, which regulated tracheal relaxation through the cAMP signaling pathway (102, 103).

**Figure 4:**
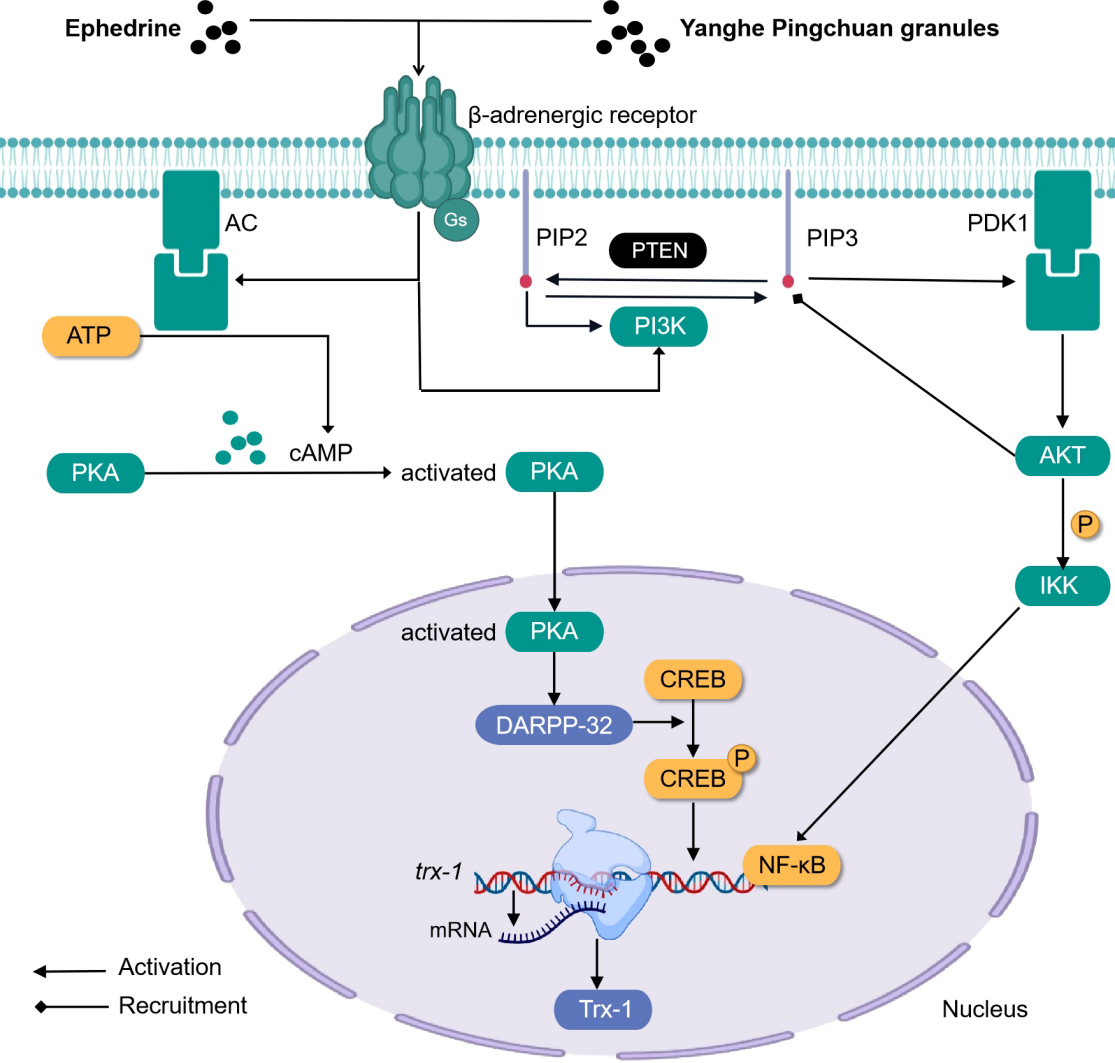
Simplified schematic diagram of cellular endocrine mechanism of some traditional Chinese medicine monomers and prescriptions for the treatment of asthma.

Ephedrine (formula: C_10_H_15_ON) (**Figure 3**) activates adrenergic receptor, a receptor that is coupled to the g protein, increases cAMP, and activates PKA. At this time, DARPP-32 was induced to be express by activated PKA, while CREB was phosphorylated by activated PKA. In the nucleus, P-CREB may bind with Trx-1 gene and initiate Trx-1 expression. Trx-1 can protect the lung tissue from injury (104). This discovery closely links ephedrine and dopamine and cAMP-regulated together in mechanism and provides a new direction for TCM monomers to participate in the β-adrenergic 2 receptor/cAMP/PKA/dopamine- and cAMP-regulated phosphoprotein signaling pathway (**Figure 4**).

### Epinephrine (adrenaline)

Epinephrine (EPI) is a catecholamine hormone secreted by the adrenal medulla after the stimulation of the sympathetic nervous system (105, 106). It is first formed by norepinephrine formed by chromic cells in the medulla and finally formed by phenylethanolamine N-methyl transferase methylation (107, 108). Adrenergic receptors (ARs) are a member of the G protein-coupled receptor family, which are divided into two families: α receptors (α1 and α2) and β receptors (β1, β2, and β3) (109, 110).

Airway remodeling is closely related to the abnormal proliferation and migration of ASMCs (111–113). Earlier studies indicated that respiratory tract cells begin to proliferate and differentiate abnormally, and the expression of PIP2, PIP3, PI3K, and AKT increases, that is, the activity of the PI3K pathway is enhanced during the occurrence of asthma. PIP3 is equivalent to a carrier, and after binding with AKT, the two can be recruited to the cell membrane, and the PDK1 on the membrane will react continuously when it meets the PIP3 carrying AKT. First, the PDK1 will be activated when it touches the PIP3, and then the PDK1 that has been activated can trigger the activation of the AKT. In addition, PIP2 participates in activating PI3K. After treatment with Yanghe Pingchuan granules, the activity of the PI3K pathway decreased significantly (114) (**Figure 4**).

When asthma occurs, the expression of EPI and AR was decreased. The expression level of both of them is closely related to the function of the hypothalamic–pituitary–adrenal (HPA) axis (115). From the perspective of TCM, in the five phases theory, the lung belongs to metal, and the kidney belongs to water. Metal and water are a mother–child relationship, so the lung and the kidney are in a mother–child relationship. In physiological function, the lungs and kidneys cooperate with each other and influence each other, which is referred to by the phrase “lung and kidney are mutually engendering”. Asthma is thought to be caused not only by the lungs but also by the kidneys (116). Kidney yang vacuity (117) is one of the pathogeneses of asthma. Kidney yang is the foundation of yang qi, and asthma patients are usually lacking congenital endowment, thus repeatedly feeling exogenously cold evil. It is easy to make kidney yang deficiency; kidney deficiency yang failure is unable to absorb qi, thus qi comes out of the lungs, causing asthma. Lung–kidney qi vacuity (118) is also one of the pathogeneses of asthma. Lung qi deficiency leads to insecurity of the interstices, which is easy to be invaded by external evil and which blocks lung collaterals; qi is disadvantageous, and fluid condenses into phlegm. Kidney qi is weak and cannot evaporate fluid, which is phlegm and accumulates into a drink. With phlegm and drink accumulation, if the human body congenital endowment is insufficient or acquired a long-term illness such as cough and asthma, then it is easy to cause asthma.

In modern medicine, the role of neuroendocrine-related HPA axis in the pathogenesis of asthma also explains this point indirectly. After treatment with Yanghe Pingchuan granules, the expression of EPI and AR was increased significantly. It suggests that Yanghe Pingchuan granules can stimulate the function of the HPA axis and improve the symptoms of kidney yang deficiency, the structural changes of airway wall, and the pathological changes of bronchus and smooth muscle (114). After its determination, it was found to contain five active ingredients (ferulic acid, sinapine thiocyanate, auercetin, acteoside, and schisandrin), providing a new idea for traditional Chinese medicine monomer in the treatment of asthma.

**Figure 5:**
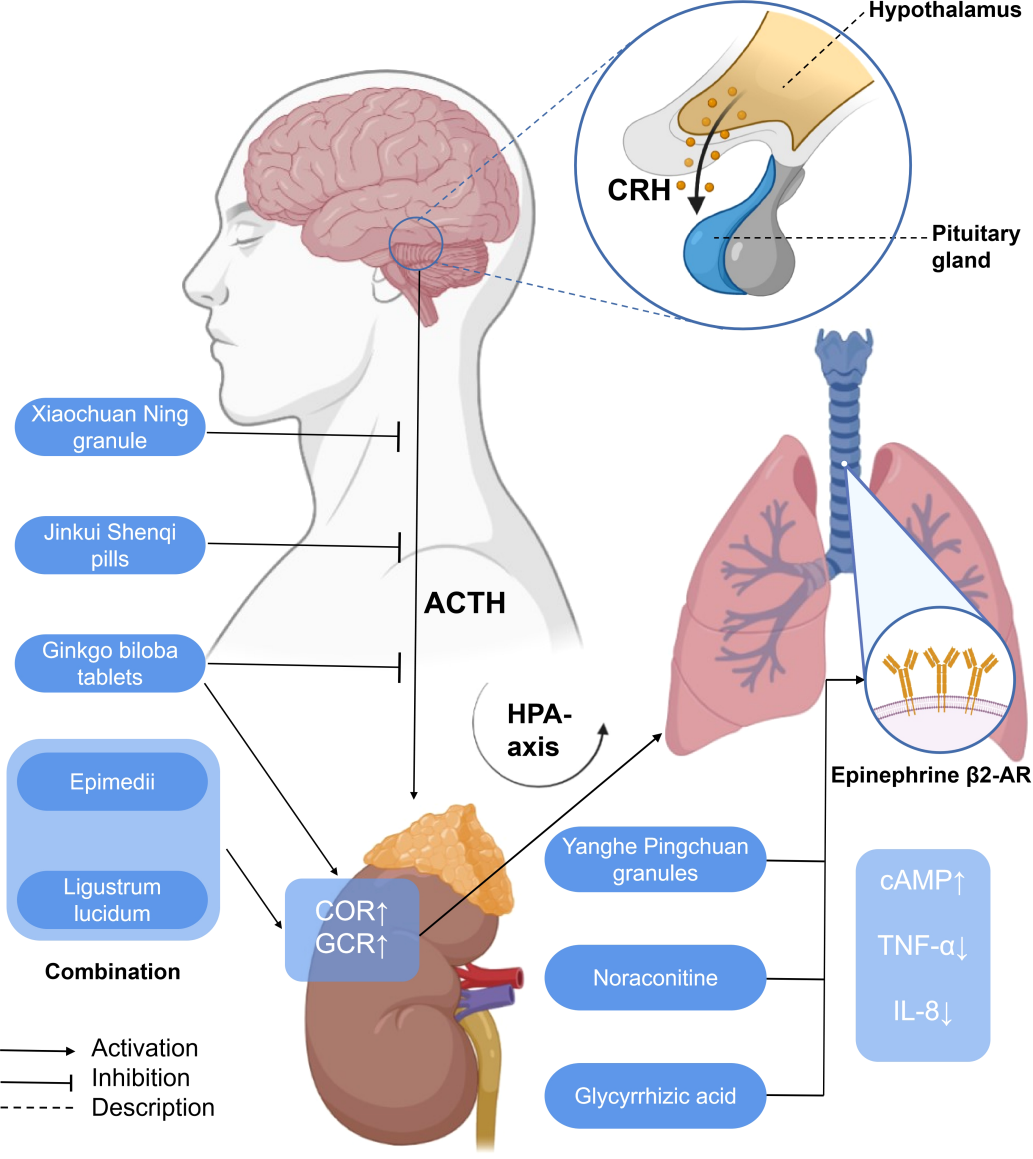
A simplified schematic diagram of the messenger role of some hormones in the treatment of asthma with traditional Chinese medicine. Yanghe Pingchuan granules, noraconitine, and glycyrrhizic acid induce airway smooth muscle dilation and relieve asthma by activating adrenaline β 2-AR. Activating G-protein-coupled cAMP signaling pathway (not shown) inhibits IL-8 secretion and asthma airway inflammation associated with NF-κB (not shown) induced by TNF- α. *Ginkgo biloba* tablets combined with Herba Epimedii and *Ligustrum lucidum* can promote the increase of COR, affect the HPA axis through the action of GCR, and alleviate airway inflammation. *G. biloba* tablets, Xiaochuan Ning granule, and Jinkui Shenqi pills can reduce the expression of ACTH, affect the HPA axis, and, finally, alleviate airway inflammation. HPA axis, hypothalamic–pituitary–adrenal axis; CRH, corticotropin-releasing hormone; ACTH, adreno-cortico-tropic-hormone; COR, cortisol; GCR, glucocorticoid receptor; cAMP, cyclic adenosine monophosphate; TNF-α, tumor necrosis factor-α; IL-8, interleukin-8.

EPI β 2-AR also functions in the treatment of asthma. Noraconitine (formula: C_9_H_10_ON_2_) (**Figure 3**) extracted from aconite can induce airway smooth muscle dilation and relieve asthma by activating epinephrine β 2-AR. Glycyrrhizic acid (formula: C_42_H_62_O_16_) (**Figure 3**) extracted from licorice is also called glycyrrhizin, and its structure is similar to that of adrenocorticotropin. When β 2-AR agonist binds β 2-AR on the cell surface, it activates G protein-coupled cAMP signal pathway, inhibits IL-8 secretion, and significantly inhibits NF-κB-related airway inflammation induced by TNF-α (119) (**Figure 5**).

It has been confirmed that immunoglobulin E (IgE) plays a certain role in the occurrence of allergic asthma.

### Eosinophil infiltration and IgE secretion

Th2 cell chemotaxis stimulates B lymphocytes to secrete a large amount of IgE and stimulates eosinophils and mast cells to produce a variety of inflammatory interleukins (120). A clinical study shows that modified Mahuang Fuzi Xixin decoction may achieve the purpose of treating bronchial asthma by reducing the content of serum IgE (121).

### Glucocorticoid and cortisol

GC is a kind of steroid hormone secreted by the adrenal cortex (122). Cortisol (COR), also known as hydrocortisone, is an adrenocortical hormone extracted from the adrenocortical cortex that has the strongest effect on carbohydrate metabolism. It is also a steroid hormone, a kind of glucocorticoid. Glucocorticoid receptor (GCR), GC, and COR are involved in the cellular endocrine mechanism of preventing immune inflammatory diseases such as allergic asthma (123).

*Ginkgo biloba* tablets combined with conventional therapy may regulate the HPA axis, upregulate the plasma COR, and reduce the level of GCR so as to reduce the hormone dependence of patients with asthma and hinder the further development of asthma (124). The combination of Herba Epimedii and Ligustrum lucidum can reduce the hormone dependence of asthmatic rats, significantly upregulate the levels of COR and GCR, affect the HPA axis through glucocorticoid action, and alleviate airway inflammation (125).

From the perspective of TCM, the liver qi ascends counterflow to the lung, which impairs depurative downbearing of the lung. More qi rise and less fall, resulting in inverted qi, cough, and even asthma (126, 127). The neurobiological mechanism of liver function of smoothing qi flow is related to the brain–gut axis (128) and the HPA axis. The dysfunction of the HPA axis and the imbalance of airway immune inflammation are the important pathological bases of psychological stress asthma. GCR is the link between the two. Xiaochuan Ning granule may regulate the function of the HPA axis, increase the expression of GCR in the lungs, and decrease the level of COR so as to restore Th1/Th2 balance and reduce airway inflammation, thus playing a role in the treatment of psychological stress asthma. Xiaochuan Ning granule may regulate the function of the HPA axis, increase the expression of GCR in the lungs, and decrease the level of COR, so as to restore Th1/Th2 balance and reduce airway inflammation, thus playing a role in the treatment of psychological stress asthma (87) (**Figure 5**).

### Corticotropin-releasing hormone and adrenocorticotropic hormone

Corticotropin-releasing hormone (CRH) is a 41-amino-acid peptide which mainly promotes the synthesis and release of adrenocorticotropic hormone (ACTH) in adenohypophysis (129). With a similarity to CRH, ACTH is also a polypeptide hormone, and its production and secretion are directly regulated by hypothalamic corticotropin-releasing factor (CRH). Excessive production of ACTH can, in turn, weaken the activity of the pituitary and hypothalamus (130). Both CRH and ACTH, which are related to the activation of central and sympathetic nerves, activate the HPA axis related to neuroendocrine (131). *Ginkgo biloba* combined with routine therapy can reduce the dependence on hormones in patients with asthma, which may regulate the HPA axis, downregulate the level of adrenocorticotropic hormone, and improve the pulmonary function indexes such as FEV1, FVC, FEV1/FVC%, and so on. The combination of Herba Epimedii and Ligustrum lucidum can inhibit the increase of ACTH, affect the HPA axis through the action of glucocorticoid, and relieve airway inflammation and airway hyperreaction (125). The Xiaochuan Ning granule may reduce the infiltration of inflammatory cells dominated by lymphocytes and the degree of airway remodeling in psychological stress asthmatic rats by reducing the levels of CRH and ACTH, regulating the activity of the HPA axis, and restoring the balance of Th1/Th2 (86). Jinkui Shenqi Pills cure and control asthma by reducing the levels of ACTH, reversing the imbalance between Th1 and Th2 cytokines, and improving the enhancement of the function of the HPA axis, which reduces the damage of immune cells and their components to the tissue structure and function of the body (87) (**Figure 5**).

### Gastrin

Gastrin (Gas) is not only a gastrointestinal hormone but also a peptide hormone. The precursor of 101 amino acids (proprogesterone) is first synthesized in the G cells of the gastric antrum, and gastrin-34, gastrin-17, and gastrin-14 are produced after processing (132). Together with gastric peptides and somatostatin, it is the main regulator of gastric acid secretion, and its effect is mainly mediated by cholecystokinin B, a G-protein coupled receptor on intestinal chromaffin cells and gastric parietal cells (133). The disorder of Gas levels in serum and cells is positively related to the dysfunction of the spleen and stomach, so it can be used as an objective index for the diagnosis and curative effect evaluation of spleen deficiency syndrome (134). From the perspective of TCM, in the five phases theory, the spleen belongs to earth, and the lung belongs to metal. Earth and metal are mother–child relationship, so spleen and lung are mother–child relationship (135). This theory was later followed up by the very interesting therapeutic approach “banking up earth and engendering metal, invigorating the spleen to benefit the lung” and confirmed by an effective pharmaceutical formulation (136). Modern studies have found that the intestinal microenvironment is closely related to spleen deficiency syndrome (137). Wenyang Pingchuan Fang can reduce the inflammatory response of asthma by increasing the level of Gas in serum, reducing the levels of IgE, TNF- α, NGF, and TrkA, alleviating the dysfunction of the spleen and stomach, and reducing the level of other inflammatory cytokines (138).

Gastrin-releasing peptide (GRP) is associated with Gas, which is synthesized by pulmonary neuroendocrine cells and can mediate airway hyperresponsiveness and airway inflammation in mice (139). At present, the relationship between TCM in the treatment of asthma and the cellular endocrine level of GRP needs to be further explored.

### Prostaglandins

Prostaglandin (PG) is a fatty acid derivative produced by the enzymatic metabolism of arachidonic acid, an unsaturated fatty acid. Prostaglandin D2 (PGD2) is a kind of PG, which is produced by the isomerization of unstable intermediate PGH2 catalyzed by prostaglandin D synthase (PGDS). As a class of pro-inflammatory cytokines, PGD2 is the main mast cell-derived prostaglandin, which responds to IgE-mediated activation (140) and then affects the development of allergic diseases such as asthma. Prostaglandins signal through G protein-coupled receptors (141). The receptor through which PGD2 signals pass is called D-prostaglandin (140). TRPVs (including TRPV1–4) are ion channels distributed in airway smooth muscles and airway epithelial cells, which regulate the intracellular calcium homeostasis. They are responsive to temperature and known as thermosensitive TRPV channels (142). External stimuli such as temperature, smoke, and chemical substances can activate the channels to open the cation influx represented by Ca^2+^ (143), which mediates the release of PGD2 and NGF and promotes the occurrence of airway hyperresponsiveness and chronic inflammation. TRPV2 is a member of transient receptor potential channels (TRPVs), which is highly homologous to TRPV1 (144). The results showed that the levels of IL-4, IL-10, NGF, and PGD2 in Balf were significantly decreased in the high- and low-dose Sanao decoction group, which was correlated with TRPV2 channel activation in lung tissue (145).

## Neuroendocrine-related signaling protein acts as messenger in the treatment of asthma with TCM

### MMP-9

Matrix metalloproteinases (MMPs) are the largest proteolytic enzymes in the matrix metalloproteinase family and are involved in tissue remodeling, wound healing, and inflammation. Matrix metalloproteinase-9 (MMP-9) is secreted from the cell to the outside of the cell in the form of Zymogen. It is activated by a series of cascades of proteases to form type IV collagenase and finally becomes an active MMP with hydrolytic ability. Its main function is to degrade and reshape the airway epithelial cells of the extracellular matrix (ECM) through abnormal production of ECM, MMP chemokines, and growth factors to participate in airway inflammation and remodeling (146, 147).

By inhibiting the expression of MMP-9, α-SMA, and TIMP-1, *Eriobotrya japonica* leaf water extract reduces the infiltration of inflammatory cells, improves the histopathological structure of lung tissue, and normalizes the intestinal flora to some extent, and it is speculated that there is a correlation between the improvement of pulmonary inflammation and the improvement of large intestinal flora (148). Shegan Mixture reduces the role of IL-17 and MMP-9 in airway remodeling and airway inflammation by reducing neutrophil activation and the degradation of collagen and promoting epithelial cell migration (149). Excessive oxidative stress induces the activation of NF-κB, which eventually leads to the overexpression of matrix metalloproteinase-9 (MMP-9) and airway remodeling (150, 151). MMP-9 also exacerbates airway inflammation because it induces an increase in inflammatory cytokines and chemokines (152). The Nrf-2/HO-1 pathway is also closely related to inflammation, oxidative stress, and apoptosis in asthma (153, 154). Cohosh extract (CRE) contains five kinds of traditional Chinese medicine monomers: caffeic acid, ferulic acid, isoferulic acid, cimicioic acid B, and cohosh acid F. CRE inhibits MMP-9 expression, activates the Nrf2/HO-1/NQO1 signal pathway, inhibits NF-κB phosphorylation, reduces the recruitment of inflammatory cells in peribronchial and perivascular lesions, reduces airway mucus secretion, inhibits eosinophil proliferation and airway hyperresponsiveness, and significantly weakens oxidative stress in asthma (155, 156). The modified Liuan decoction downregulates the gene expression of MMP-9 and TIMP-1, a specific inhibitor of MMP-9 and MMP-9, in the lung tissue of rats by inhibiting the expression of MMP-9, regulates the balance of MMP-9/TIMP-1, reduces ECM deposition, and inhibits airway remodeling (157). Earthworm extract can effectively inhibit the expression of MMP2, MMP9, and TIMP-1 protein in lung tissue, reduce the total number of cells and the number of eosinophils, macrophages, lymphocytes, and neutrophils in BALF, and then inhibit airway inflammation and airway remodeling in asthmatic mice. The mechanism may be related to the inhibition of the Th2 immune pathway by regulating Th1/Th2 balance (158).

### Sema4D

Semaphorin4D (Sema4D), also known as CD100, like semaphorins4A (Sema4A) (159), is a member of the semaphorin family and an IV member of the signaling glycoprotein family (160). It has long been considered as a brain signal protein. Recent studies have found that it participates in neuroendocrine processes, such as the long-term inflammation of endogenous or exogenous antigens, and plays a prominent role in the immune system, nervous system, tumor invasion, and metastasis. Sema4D can regulate T-cell initiation and stimulate macrophages, DC, NK cells, and neutrophils (161).

Sema4D may inhibit the release of endocrine hormones in the HPA axis by stimulating the release of SP and the expression of c-fos protein in rat brain, thus aggravating asthma. Middle- and high-dose Kechuanning oral liquid can regulate Sema4D to reduce the c-fos protein expression and SP content, increase the CRH content and ACTH content, and indirectly improve the inhibition of Sema4D on the HPA axis in asthmatic rats (162). Kechuanning oral liquid can also downregulate the expression of Sema4D in lung and spinal cord tissue, inhibit TNF-α to stimulate eosinophils and mast cells, then inhibit the secretion of IL-6, promote Th1 and inhibit Th2 differentiation so as to regulate the balance of Th1/Th2, and then inhibit inflammatory factors such as IL-8, IL-4, and TNF- α. IFN-γ returns to the original level, thus reducing airway inflammation (163, 164). In addition, Kechuanning oral liquid can also regulate the expression of Sema4D—on the one hand downregulating the expression of α-SMA and MMP-2 in the lung tissue of asthmatic rats, reducing collagen deposition, and improving airway remodeling (165); on the other hand, Sema4D combines with Sema4D receptors CD72 and Plexin-B1, inhibits the PI3K/Akt pathway, then affects airway inflammation and airway remodeling, and achieves a therapeutic effect (166).

### Nerve growth factor

Nerve growth factor is a high molecular weight polypeptide and one of the earliest neurotrophic factors (neurotrophin) (167). It can not only exert biological activity on the central and peripheral nervous system by means of target cell secretion, autocrine or paracrine, but also regulate a variety of allergic inflammatory effector cells (eosinophils, Th2 and mast cells) to exert biological activity on the immune system (168), thus playing a role in asthma inflammation such as airway remodeling and bronchial hyperresponsiveness (169).

NGF is involved in the regulation of neural differentiation in asthma. It can induce nerve cells to produce tachykinin and upregulate the expression of its receptors, which, in turn, induces neurogenic inflammation and promotes airway hyperresponsiveness. The Buzhong Yiqi decoction can reduce the level of NGF in serum and then reduce the level of IL-4 and eosinophil in serum, which further proves the mechanism of anti-airway inflammation of the Buzhong Yiqi decoction from the level of molecular biology (170). TrkA is the receptor tyrosine kinase of NGF. Together with TNF-α, they are both important initiators in the inflammatory process of asthma, which can activate NGF-mediated inflammatory signaling pathways. Wenyang Pingchuan Fang can increase the level of Gas in the serum of asthmatic mice with spleen yang deficiency syndrome, reduce the level of TrkA, TNF-α, and NGF, and reduce the level of inflammatory cytokines so as to reduce airway inflammation (138). The San’ao decoction can activate TRPV2 channels distributed in airway smooth muscle and airway epithelial cells, reduce the content of IL-4, IL-10, and NGF in BALF, and balance the inflammatory response of other cells through NGF (145). The main functional components of the Maxing Shigan decoction are ephedrine, pseudoephedrine, methyl ephedrine, amygdalin, L-amygdalin, glycyrrhizin, glycyrrhizic acid, and licorice flavonoids. It can downregulate TRPV1 protein, reduce the content of NGF in lung tissue, then reduce the infiltration of pulmonary intercellular inflammatory cells, and reduce airway hyperreaction (171).

### Mitochondrial calcium uniporter

The mitochondrial calcium uniporter (MCU) is a channel protein responsible for mitochondrial matrix Ca^2+^ uptake, is an important molecular machine for mitochondrial calcium uptake, and plays a key role in mitochondrial energy metabolism and the maintenance of cellular calcium homeostasis (172). The absorption of calcium by mitochondria is not simply transported by a single protein but by a complex formed by multiple proteins. MCU is in the middle (173) of this protein complex, which can inhibit autophagy and reduce inflammation *in vitro* and *in vivo* (174). In the presence of allergens, MCU leads to mitochondrial dysfunction and increased production of ROS, which, in turn, leads to the loss of barrier function of airway epithelial cells and the increase of autophagy level, finally leading to apoptosis (175). This suggests that MCU acts as a messenger to regulate the process of mitochondrial matrix Ca^2+^ uptake to affect the activity of epithelial cells in allergic asthma and maintain the barrier function of airway epithelial cells, thus affecting the progression of asthma (176). In addition, MCU can reduce the relative expression of caspase-3, regulate the activity of caspase-3, protect the mitochondrial membrane potential, prevent apoptosis, and exert additional effects (177).

Shang-Huang-Lian inhibited the levels of tIgE, IgE, and mMCP-1 induced by shrimp protein sensitization and decreased the production of Th2 cytokines (such as IL-4, IL-5, and IL-13) in BALF. It inhibits basophil activation (178) by activating MCU, thereby preventing Th0 cells from differentiating into Th2 types and stabilizing mast cells and thus reducing inflammatory cell infiltration and airway smooth muscle thickness. In addition, a single administration of Shuang-Huang-Lian can directly activate MCU to enhance mitochondrial calcium uptake and reduce the level of free calcium in the cytoplasm, thus preventing mast cell degranulation (179).

## Discussion

Overall, this review highlights the recent research advances in the pathogenesis and treatment of asthma under cellular endocrinology and provides an overview of TCM research related to this topic. Over the years, it is recognized that the role of dendritic cells, eosinophils, T lymphocytes, and various cytokines in the pathogenesis of asthma has become increasingly evident. On this basis, it is gradually recognized that hormones, pulmonary neuroendocrine cell secretions, and neuroendocrine signal proteins act as intercellular messengers to affect immune cells and various cytokines.

Asthma is a proinflammatory disease with a lot of attention on IgE production and B cell, T follicular helper (TFH) cell subsets, follicular regulatory T (TFR) cell, and other helper T cell participation. Studies show that TFR cell and TFH cell subsets (TFH2 cells, TFH13 cells, and TFR cells) and their iconic cytokines IL-21 have been proved to be related to the production of IgE in asthma to a large extent (180–182). At present, traditional Chinese medicine can regulate inflammatory and autoimmune diseases (183), but the effect of TFR cell and TFH cell subsets on the production of specific IgE by regulating hormones, pulmonary neuroendocrine cell secretions, and neuroendocrine signal proteins remains to be further studied.

As one of the main treatments of complementary and alternative medicine, TCM has a long history in the treatment of asthma. The Yellow Emperor’s Inner Canon and other ancient medicine books of TCM provide a theoretical basis for the treatment of asthma: “lung and large intestine stand in interior–exterior relationship”, “lung and kidney are mutually engendering”, “liver governs upbearing, lung governs downbearing”, “banking up earth and engendering metal” (**Table 1**). Prescription (**Table 2**), traditional Chinese medicine monomer, traditional Chinese medicine monomer complex, single herbs (**Table 3**), and proprietary Chinese medicine (**Table 4**) for the treatment of asthma have small side effects, diverse structures, a wide range of sources, and have a multi-target synergistic effect.

**Table 1:** Corresponding therapeutic drugs and biochemical indexes of asthma from the perspective of cellular endocrine and basic theory of traditional Chinese medicine (TCM).

Relevant basic theory of TCM	Physiological structure/environment	Signaling pathway	Drug	Biochemical indexes	Reference	Theoretical basis	Experimental basis
Lung and large intestine stand in interior–exterior relationship (肺合大肠)	Lung and intestinal axis; intestinal microenvironment	TLR/NF-κB signaling pathway	Mirabilite, Huatan Huoxue formula	Lung tissues: SP, VIP, NK-1R, NKA, NKB, IL-25 m RNA, Sphk1 m RNA, iILC2s Intestinal tissue: SP, VIP, NK-1R, NKB, IL-25 m RNA, Sphk1 m RNA, S1PR1, ILC2s, iILC2s, n ILC2s Stomach tissues: SP, VIP, NKB Heart tissues: VIP Spleen tissues: NKB Serum: Ig E BALF: IL-4, IL-9, IL-13, IL-25, IL-33, TSLP, IL-6, AREG, ILC2s, mi R-155, mi R-146a	The Yellow Emperor’s Inner Canon (黄帝内经)	(68) Fu et al., 2018 (71) Dang et al., 2019	(67) Zhong et al., 2013 (78) Fu et al., 2020
Lung and kidney are mutually engendering (肺肾同源)	Hypothalamic–pituitary–adrenal axis	cAMP/TNF-α/NF-κB signaling pathway	Yanghe Pingchuan granules, Noraconitine, Glycyrrhizic acid, Modified Mahuang Fuzi Xixin decoction, Jinkui Shenqi Pills	Bronchial tissue: imbalance between Th1 and Th2 cytokines, P13K, AKT, PIP2, PIP3, PCNA, IL-6, IL-8, IL-1β HPA axis function: EPI, AR, ACTH Serum: Ig E, imbalance between Th1 and Th2 cytokines, ACTH, IFN-γ, IL-4	The Yellow Emperor’s Inner Canon (黄帝内经)	(115) Fitzgerald et al., 2021 (116) Kong et al., 2017 (117) Shen et al., 2012 (118) Dong et al., 2015	(114) Pan et al., 2018 (119) Yang et al., 2009 (121) Gan et al., 2020 (87) Ji et al., 2020
Liver governs upbearing, lung governs downbearing, (肝宣升, 肺宣降)	Hypothalamic–pituitary–adrenal axis; brain–gut axis	————	Xiaochuan Ning granule	Serum: CRH, ACTH, COR, IL-4, IL-7 BALF: CRH, ACTH, COR, IL-4, IL-7 Lung tissue: IFN-γ, GR	The Yellow Emperor’s Inner Canon (黄帝内经)	(126) Jiang et al., 2020 (127) Shao et al., 2019 (128) Li et al., 2010	(86) Li et al., 2021
Banking up earth and engendering metal (培土生金)	Intestinal microenvironment	————	Wenyang Pingchuan Fang	Serum: gas, TNF-α BALF, IgE, NGF, TrkA	The Yellow Emperor’s Inner Canon (黄帝内经)	(135) Liu et al., 2015 (136) Lai et al., 2013 (137) Yang et al., 2021	(138) Sun et al., 2020

**Table 2:** The prescription, composition, formulation, and mechanism of asthma treatment.

Prescription name	Main components	Formulation	Indicator	Experiment subjects	Reference
BuShenYiQi formula	Epimedium brevicornu Rehmannia glutinosa Astragalus membranaceus Scutellaria Paeonia lactiflora	Decoction	Lung tissue: percentage of ILC2s ↓ Percentage of Th9 cells ↓ Expression of type 2 cytokines (IL-5, IL-13 and IL-9)↓, GATA3↓, PU.1↓, IRF4↓, VIP↓, VPAC2↑, Percentage of VPAC2+CD90+ cells↓	OVA-induced asthmatic mice	(31) Huang et al., 2021
Modified BuShenYiQi formula	Epimedium brevicornu, Astragalus membranaceus, Rehmannia glutinosa, Scutellaria baicalensis, Paeonia lactiflora	Decoction	Percentage of ILC2s and Th9 cells↓ Type 2 cytokines↓, GATA3↓, PU.1↓, IRF4↓ VIP↓, VPAC2↓ Percentage of VPAC21CD901 cells↓	OVA-induced asthmatic mice	(31) Huangi et al., 2021
The combination of Sanao decoction and Xiaochengqi decoction	Ephedra, almond, licorice, rhubarb, Fructus aurantii, Magnolia officinalis	Decoction	Serum: VIP↑, TNF-α↑, IL-6↑, Lung tissue: TNF-α↓, IL-6↓, p38 MAPKmRNA↓	OVA-induced asthmatic mice	(35) Hui et al., 2022
Modefied Xiaofeng San	Schizonepeta tenuifolia, Fangfeng, bombyx mor, Periostracum cicadae, Lumbricus, Perilla leaf, Perilla seed, Ephedra, Fried bitter almond, Magnolia officinalis, Chuanxiong, Radix Stemonae, Angelica, Roasted licorice	Powder	Pulmonary function: FEV1↑, FEV1/FVC↑, FEV1%↑ FeNo↓, Plasma: VIP↑, SP↓	Patients with chronic persistent asthma	(36) Jia et al., 2018
White mustard San	Sinapis alba, Corydalis edulis maxim, Euphorbia kansui, *Asarum*, ginger	Powder	Serum: SP↓, CGRP↓, VIP↑, lgE↓	OVA-induced asthmatic mice	(50) Wang et al., 2019
Maxing Shigan decoction	Ephedra, almonds, roasted licorice, plaster	Decoction	Lung tissue: TRPV1↓, IL-4↓, IL-13↓, PGE2↓, SP↓ IL-2↓, TNF-α↓, EGFR↓, CGRP↓, SP↓, NK-1R↓, NGF↓	Asthmatic mice induced by 30s/secondary spray of the mixture of 2% acetylcholine chloride and 0.4% histamine phosphate, OVA-induced asthmatic mice	(51) Li et al., 2022 (60) Xu et al., 2021 (171) Li et al., 2021
Yupingfeng formula	Radix Astragali seu Hedysari, Atractylodes macrocephala, Radix Saposhnikoviae	Decoction	Serum: IL-5↓, SP↓	Children with cough variant asthma	(52) Li et al., 2018
Modified Bainiu Xuanfei decoction	Fried Ephedra, *Bombyx mori*, cicada slough, Burdock, peach kernel, almond, Bupleurum, Schizonepeta tenuifolia, peppermint, Aster, Baibu, reed root, licorice, Mulberry bark	Decoction	Lung tissue: TNF-a↓, IL-8↓, IL-4↓, IL-5↓,NKA↓, SP↓, CGRP↓	Patients with cough variant asthma	(76) Ma et al., 2017
Minkeng Jian	Radix scrophulariae, Fructus Ephedrae, Fructus Schisandrae, Radix Saposhnikoviae, Uncaria, Lumbricus, Bombyx batryticatus	Decoction	Induced sputum supernatant fluid: SP↓, NKA↓ Plasma: IFN- γ↓, TNF- α↓, IL-4↓, IL-5↓	Patients with chronic persistent asthma	(77) Zhong et al., 2014
Huatan Huoxue formula	White mustard seed, Perilla, Semen raphani, Semen persicae, Flos carthami, cooked rehmannia, Chinese angelica, Radix paeoniae alba, Ligustrazine	Decoction	Serum: Ig E↓ BALF: IL-4↓, IL-9↓, IL-13↓, IL-25↓, IL-33↓, TSLP↓, IL-6↓, AREG↓, ILC2s↓, mi R-155↓, mi R-146a↑ Lung tissues: IL-25 m RNA↓, Sphk1 m RNA↓, iILC2s↓ Intestinal tissue: IL-25 m RNA↓, Sphk1 m RNA↓, S1PR1↓, ILC2s↓,i ILC2s, n ILC2s	OVA-induced asthmatic mice	(78) Fu et al., 2020
Modified Mahuang Fuzi Xixin decoction	Ephedrae Herba, Radix aconitu laterlis preparata, Asarum heterotropoides, Zingiberis rhizoma, Schisandrae chinensis fructus, Cinnamomi ramulus, Scutellaria baicalensis, Radix glycyrrhizae	Decoction	Serum: Ig E↓	Patients with mild bronchial asthma during acute exacerbation	(121) Gan et al., 2020
Buzhong Yiqi decoction	Radix Astragali seu Hedysari, Radix Codonopsis, fried licorice, Rhizoma atractylodis alba, Pericarpium citri reticulatae, Rhizoma Cimicifugae, Radix Bupleuri, Radix angelicae sinensis	Decoction	Serum: NGF↓, IL-4↓, EOS↓ Pulmonary function: FEV1↑, PEF↑	Patients with chronic persistent asthma	(170) Tang et al., 2018
Wenyang Pingchuan Fang	Fried ephedra, Bitter apricot seed, Fructus Perillae, Pericarpium Zanthoxyli, Semen Persicae, Fried licorice, Sliced processed aconite, Rhizoma atractylodis alba, Radix Codonopsis, Rhizoma Zingiberis	Decoction	Serum: gas↑, TNF-α↓ BALF, IgE↓, NGF↓, TrkA↓	OVA+Irregular diet+Overwork induced asthmatic mice	(138) Sun et al., 2020
San’ao decoction	Ephedra, Semen armeniacae amarum, Radix glycyrrhizae	Decoction	BALF: IL-4↓,IL-10↓,NGF↓,PGD2↓	OVA+TMA induced asthmatic mice	(145) Zhang et al., 2020
Shegan Mixture	Ephedrine, almond, Belamcanda chinensis, Rorippa indica, Radix scutellariae, Batryticated silkworm	Decoction	Peripheral blood: IL-10↓, IL-17↓, MMP-9↓, TGF- 1↓	Patients in acute exacerbation stage of asthma	(149) Du et al., 2016
Modified Liuan decoction	Rhizoma pinelliae, Exocarpium citri rubrum, Poria, Semen armeniacae amarum, white mustard, Radix glycyrrhizae, Pumex, Semen lepidii, Fructus trichosanthis, Rhizoma arisaematis cum bile, stir-fried radish seed	Decoction	Lung tissue: MMP-9↓, TIMP-1↓	Aluminum hydroxide and OVA-induced CVA mice	(157) Du et al., 2021

**Table 3:** The mechanism of traditional Chinese medicine monomer, traditional Chinese medicine monomer complex, and single herbs in the treatment of asthma.

Species	Name	Source	Drug absorption	Indicator	Experiment subjects	Reference
Traditional Chinese medicine monomer	Menthol	Mint	Atomization inhalation	Bronchial epithelial cells: SP↓ NK-1R↓	OVA-induced asthmatic mice	(53) Wang et al., 2018 (54) Wang et al., 2018
	Ephedrine	Ephedra	Intragastric administration	cAMP↑, DARPP-32↑, CREB↑, phosphorylated PKA↑, p-CREB↑, Trx-1↑	PC12 cells of the rat	(97) Jia et al., 2013
	Noraconitine	Aconite	Intragastric administration	β2-AR↑	Tracheal smooth muscle *in vitro* model and guinea pig asthma model	(119) Yang et al., 2009
	Glycyrrhizic acid	Licorice	Intragastric administration	β2-AR↑, cAMP↑, TNF-α↓, NF-κB↓, IL-8↓	OVA-induced asthmatic mice	(119) Yang et al., 2009
	Icariin	Herba Epimedii	Intragastric administration	BALF: IL-13↓, ET-1↓, TGF-β1↓, VEGF↓ Serum: IL-13↓, ET-1↓, TGF-β1↓, VEGF↓	OVA-induced asthmatic mice	(93) Hu et al., 2019
Traditional Chinese medicine monomer complex	Caffeic acid Ferulic acid Isoferulic acid Cimicifugic acid B Cimicifugic acid F	Cimicifugae Rhizoma	Intragastric administration	MMP-9↓, Nrf2/HO-1/NQO1↑, NF-κB↓	OVA-induced asthmatic mice	(156) Lim et al., 2021
	Eriobotrya japonica leaf water extract	Eriobotrya japonica	Intragastric administration	α-SMA↓, MMP-9↓, TIMP-1↓	OVA-induced asthmatic mice	(148) He et al., 2021
	Earthworm extract	Earthworm	Intragastric administration	Lung tissues: MMP2↓, MMP9↓, TIMP-1 BALF: Eot↓, IL-4↓, IL-5↓, IL-13 Plasma: IgE↓	OVA-induced asthmatic mice	(158) Zhang et al., 2021
Single herbs	Mirabilite	————	Intragastric administration	Lung tissues: SP↓, VIP↑, NK-1R↓, NKA↓, NKB↓ Intestinal tissue: SP↑, VIP↑,NK-1R↑↑, NKB↓ Stomach tissues: SP↑, VIP↓,NKB↓ Heart tissues: VIP↑ Spleen tissues: NKB↓	OVA-induced asthmatic mice	(67) Zhong et al., 2013
	Herba Epimedii	————	Intragastric administration	HPA axis function: Serum: COR↑, Branchoalveolar lavage fluids: GCR↑, Plasma: ACTH↓	OVA-induced asthmatic mice	(125) Liu et al., 2013
	Ligustrum lucidum	————	Intragastric administration	HPA axis function: Serum: COR↑, Branchoalveolar lavage fluids: GCR↑, Plasma: ACTH↓	OVA-induced asthmatic mice	(125) Liu et al., 2013

**Table 4:** The mechanism of traditional Chinese patent medicine in the treatment of asthma.

Species	Name	Source	Drug absorption	Indicator	Experiment subjects
Huanglong cough oral liquid	Fried ephedra, Almond, Perilla, Mulberry bark, Bupleurum, Periostracum cicadae, Lumbricus, Inula japonica	Atomization inhalation	Lung tissue: LTE4↓, NGF↓, CGRP↓	Aluminum hydroxide and OVA-induced CVA mice	(52) Li et al., 2018
Yanghe Pingchuan granules	Ephedra sinica, Inula japonica, Morinda officinalis, Schisandra chinensis, Sinapis alba, Draba nemorosa, Angelica sinensis, Platycodon grandiflorus	Intragastric administration	Bronchial tissue: P13K↓, AKT↓, PIP2↓, PIP3↓, PCNA↓, IL-6↓, IL-8↓, IL-1β↓ HPA axis function: EPI↑, AR↑	OVA-induced asthmatic mice	(114) Pan et al., 2018
Ginkgo biloba tablets	Ginkgo biloba	Oral administration	Pulmonary function: FEV1↑, FEV1/FVC↑, HPA axis function: COR↑, GCR↑ (Plasma)	Patients with hormone-dependent asthma	(124) Zheng et al., 2016
Xiaochuan Ning granule	Radix Bupleur, Semen Lepidii, Whole trichosanthes, Radix Scutellariae, Rhizoma Pinelliae, Ramulus Uncariae Cum Uncis, Saposhnikovia, Radix Paeoniae Alba, Radix Peucedani, Fried ephedra, Lumbricus, Radix Salviae, Miltiorrhizae	Intragastric administration	Serum: CRH↓, ACTH↓, COR↓, IL-4↓, IL-7↓ BALF: CRH↓, ACTH↓, COR↓, IL-4↓, IL-7↓ Lung tissue: IFN-γ↓, GR↑	28-day stress stimulation and OVA-induced asthmatic mice	(129) Li et al., 2021
Jinkui Shenqi Pills	Processed aconite, Cassia twig, Rehmannia, Dioscorea root, Cornus fruit, Alisma, Poria, Cortex of the Peony Tree Rote	Intragastric administration	BALF: imbalance between Th1 and Th2 cytokines↓ Serum: imbalance between Th1 and Th2 cytokines↓, ACTH↑, IFN-γ↑, IL-4↓ HPA axis function: ACTH↑ (Serum)	OVA-induced asthmatic mice	(87) Ji et al., 2020
Kechuanning oral liquid	Fried ephedra, Bitter apricot kernel, Raw gypsum, Folium Isatidis, Radix Astragali seu Hedysari, Semen Persicae, Tea bust, Radix Glycyrrhizae	Intragastric administration	Serum: IL-4↓, IL-6↓, IL-8↓, TNF-α↓, IFN-γ↑ BALF: IL-4↓, IL-6↓, IL-8↓, TNF-α↓, IFN-γ↑ Lung tissue: Sema4D↓, MMP-2↓, α-SMA↓ Spinal cord tissue: Sema4D↓	OVA+Hep-2+0.9%NaCl induced asthmatic mice	(162) Ji et al., 2018 (163) Chen et al., 2020 (164) Wu et al., 2020 (165) Chen et al., 2019
Shang-Huang-Lian	Flos Lonicerae, Fructus Forsythiae, Radix Scutellariae	Intragastric administration	BALF: tIgE↓, IgE↓, mMCP-1↓, Th2 cytokines↓IL-4↓, IL-5↓, IL-13↓, eosinophils↓ MLN cells: Th2 cytokines↓	Shrimp protein (SP)-induced mice	(178) Gao et al., 2019

The clinical and experimental research of TCM in the treatment of asthma has been conducted, and some meaningful results have been achieved, but some limitations still exist. In terms of the source of ingredients, most of the Chinese herbal ingredients extracted by researchers cannot verify the purity of the compounds, and the effects of the extraction methods on the chemical and physical properties of the components are not reflected in the experimental design. In terms of side effects, some animal experiments lack consideration of the toxic and side effects of traditional Chinese medicine in the treatment of asthma. In terms of study subject, gastrointestinal hormones play an increasingly important role in the treatment of asthma in recent years, the research on it is not systematic enough, and the cellular and molecular mechanisms involved are not clear enough, which is worthy of further study from the perspective of cellular endocrine. In terms of drug administration, drug inhaler is a safe, efficient, and relatively cheap method for the treatment of asthma, but most of the traditional Chinese medicine studies focus on intragastric administration, and few studies evaluate the administration of nasal drops. This can be used as a new direction of the next stage of research.

## Data availability statement

The data used to support the results of this study are included in the article.

## Author contributions

ZM, HC, and CD contributed equally to this work. All authors contributed to the article and approved the submitted version.

## Funding

This work was supported by COVID-19 Emergency Response Project of Shanghai Sixth People's Hospital in 2022 (ynxg202218) the Project of Shanghai Science and Technology Commission (19401970600) for the Project of Shanghai Science and Technology Commission (19401932500), and Shanghai will further accelerate the 3-year action plan for the development of TCM (2018–2020) for major clinical research on TCM [ZY (2018–2020)-CCCX-4010], the Innovation Fund of Integrated Traditional Chinese and Western Medicine, School of Medicine, Shanghai Jiao Tong University (18zxy002), the 2019 Teacher Training and Development Project of Medical School of Shanghai Jiao Tong University (JFXM201909), the Experimental Project of Scientific and Technological Innovation for College Students of Heilongjiang University of Traditional Chinese Medicine (16041200019), and the Innovation and Entrepreneurship Training Programme for Students of Heilongjiang University of Chinese Medicine (X202110228007) and National General Project of Innovation and Entrepreneurship training Program for College students in Heilongjiang Province (202210228074) and Provincial General Project of Innovation and Entrepreneurship training Program for College students in Heilongjiang Province (S202210228075).

## Acknowledgments

The author thanks Heilongjiang University of Chinese Medicine and Shanghai Jiao Tong University Affiliated Sixth People’s Hospital for their support.

## Conflict of interest

The authors declare that the research was conducted in the absence of any commercial or financial relationships that could be construed as a potential conflict of interest.

## Publisher’s note

All claims expressed in this article are solely those of the authors and do not necessarily represent those of their affiliated organizations, or those of the publisher, the editors and the reviewers. Any product that may be evaluated in this article, or claim that may be made by its manufacturer, is not guaranteed or endorsed by the publisher.
